# Immunitas magistra vitae: The time has come to replace humanity’s teacher of life

**DOI:** 10.1038/s44319-025-00592-5

**Published:** 2025-10-27

**Authors:** Vladimir Leksa

**Affiliations:** Laboratory of Molecular Immunology, Institute of Molecular Biology, Slovak Academy of Sciences, Bratislava, Slovakia

**Keywords:** Economics, Law & Politics, History & Philosophy of Science, Immunology

## Abstract

Humankind apparently has forgotten the lessons from the past and finds itself in deadly dangers. Following the rules by which a well-balanced immune system works could save us.

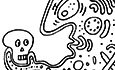

“*Historia magistra vitae*,” says an old Latin proverb: *“History is the teacher of life.”* Now, what has history really taught us? Or, put differently, what have we learned from history? Apparently, nothing. We do not need to look far for proof: just look at the previous century, which taught us some very hard lessons. And yet, we failed to learn from them.

“Now, what has history really taught us? Or, put differently, what have we learned from history?”

## Totalitarianism regained

The 20th century saw the rise of two totalitarian systems: fascism and communism. Fascism was “invented” in Mussolini’s Italy but copied and blown up to monstrous proportions by Nazi Germany. Communism first took root in Russia and was exported to many other countries. Both systems cost hundreds of millions of lives, provoked multiple wars—the Second World War, the Korean War, the Vietnam War, the Cold War—the occupation of many states, and the subjugation of their people under totalitarian regimes. How could such murderous dictatorships arise, seize power, and thrive in Europe, the cradle of Athenian democracy, Iceland’s democratic tradition, and the division of power and human rights?

The bait offered to people by dictators plays a key role in the rise of evil. Its basic ingredients were greed, envy, and hatred; and fear among those who did not fall for these lures. Nevertheless, a significant share of the blame goes to philosophers, scientists, artists, and politicians elsewhere, who through their silence, loyalty, or naivety, ceded space to evil. In their defense, it must be said that they had no experience, no lessons from history about how to confront such a systematic and absolute form of evil.

But alas! Barely a century later, a hybrid of these murderous systems—fascism and communism—is spreading like a hydra across the world; and is taking hold in countries that seemed resistant a hundred years ago. History, it seems, has still taught us nothing. But today, we have no excuses.

## Defensibility lost

Yet, the 20th century was not only the era of war and genocides, but also of great art and great science. It was the era during which humanity, for the first time, succeeded in completely eradicating infectious diseases that had killed billions throughout history; thanks to vaccination.

The principle of vaccination was first recorded in ancient China (Weiss and Esparza, 2015) when healers applied the contents of pustules from children sick with smallpox to the skin of healthy children; or blew powder from dried scabs into the nostrils of healthy children; or dressed healthy children in the shirts of the sick. Rumors of this method—variolation—reached Europe via the Silk Road, where local doctors adopted it. Among them was Slovak Ján Adam Rayman, a pioneer of variolation in Central Europe, who in 1721 successfully inoculated his own child and others; and published a report in the scientific literature (Bartunek, 1990).

Although variolation represented significant progress in protection against smallpox, it also carried a great risk of infecting otherwise healthy people with the causative agent, variola virus. A safer technique—vaccination—was developed by Edward Jenner at the end of the 18th century, who applied the related but harmless vaccinia virus as an immunizing agent. This moment marked the beginning of the history of immunology—the science of the body’s defenses. In less than 200 years, Jenner’s discovery of vaccination led humanity to the complete eradication of smallpox. Until some time ago, it seemed that other infectious diseases—such as polio, measles, wand hooping cough—would meet the same fate.

But alas! Barely half a century after the last recorded smallpox case (Meyer et al, 2020), we face an unprecedented rise in deaths from measles.

## The great lapse of memory

In 2024, Europe saw the highest number of measles cases in over 25 years, double the number from 2023. In the Americas, the number of cases in early 2025 increased fourfold compared to the same period in 2024 (Do and Mulholland, 2025). This drastic rise in measles cases is primarily driven by declining vaccination rates and increased vaccine hesitancy. More than half of the reported cases worldwide required hospitalization, and most deaths occurred among unvaccinated children. Children with a weakened immune system who can not get the vaccination, such as those who have undergone bone marrow transplants after cancer, have the highest risk of dying. The decision of parents not to vaccinate their children thus directly endangers not only their own offspring but also the children of others.

Our current civilization seems to have lost its memory. We voluntarily elect populists from the ranks of those who, a few decades ago, oppressed and persecuted our grandparents and great-grandparents. And we voluntarily lower the shields that have reliably and for long protected us from deadly diseases. The result of this neglect will be again oppression at the hands of tyrants, and deaths from measles and other diseases we thought we only knew from textbooks. We have reached a state where we can send satellite probes to comets hurtling through space or observe the movement of individual molecules in cells, while we voluntarily abandon the wisdom on which this progress is built. We open the gate to killers, whether from the realm of viral, unicellular, or multicellular beings. Where did we go wrong?

“We open the gate to killers, whether from the realm of viral, unicellular or multicellular beings. Where did we go wrong?”

## Time for some change in the school-of-life system

History as the teacher of life has obviously failed. As an immunologist, I propose that humanity choose immunity as its new teacher of life.

“History as the teacher of life has obviously failed. As an immunologist, I propose that humanity choose immunity as its new teacher of life.”

When I broke my leg as a boy, the doctor put it in a cast, and the bone healed. But the cast did not protect me from further fractures. When the doctor vaccinated me against smallpox, I was protected for life. Immunology is the only branch of medicine that can not only cure a disease but also completely eradicate it. If we choose immunity as our teacher, perhaps we will regain our memory and eradicate evil from the face of the Earth.

When Jenner performed the first vaccination at the end of the 18th century, he did not know what caused smallpox, nor did he have any idea how the vaccine protected against it. It took 200 years for us to find out. Today, we know that infectious diseases are caused by pathogenic viruses and microorganisms, and that we defend ourselves against them with highly specialized immune cells, which are born in our bone marrow. Among them are ravenous neutrophils that devour enemies on the spot; experienced macrophages that are already trained for their errand in the bone marrow; B cells that produce an arsenal of antibodies; and T cells that help others but can also directly confront infected cells. These are all very useful skills that are worth learning, are not they?

More importantly, this army has platoons of memory cells that, after the first encounter, never forget the face of an enemy, and immediately sound the alarm when it reappears. It is this immunological memory that is the reason why we fall ill with some infectious diseases only once. And thanks to this immunological memory, vaccines work.

It is important that immunity actively maintains its memory throughout our lives. We humans would certainly benefit from learning this. Hoaxes and information weapons of hybrid war would have no chance of deceiving us. But we could learn much more from immunity. Our immune cells can distinguish between our own body and strangers. And if the later do not harm us or even help us—like commensal bacteria and viruses in our intestines—immune cells tolerate them. On the other hand, if our own cells go wrong, for example, if they transform into tumor cells, immune cells can kill them.

Not only does immunity recognize what is strange and what is not, but also what is dangerous and what is harmless (Matzinger, 1994). It would definitely be worth learning too: *“Imunitas magistra vitae*”—“Immunity is the teacher of life.” Immunity is neither xenophobic nor nationalist; and immune cells that do not follow these restrictions undergo apoptosis, a programmed death. Unfortunately, it might be not necessarily so.

## Immunity as a smart home

You have surely noticed, on walks around town, how some newcomers have built their houses. Tall and thick walls behind which vicious guard dogs bark; you can not even see the house itself. And alarms everywhere. I imagine it must be quite dangerous for mail carriers to deliver a package. In the language of immunologists, this would be a strong allergic reaction—type I hypersensitivity caused by too aggressive immune cells, which escaped their restrictions. Sociologists may call it xenophobia.

In the worst case, the owner himself could become the victim. He forgets his door key, climbs in through the balcony, accidentally sets off the alarm, and the summoned police shoot him. This would be a clear autoimmune response—type II hypersensitivity; or the hunt for a perceived internal enemy based on the spread of disinformation.

Or, you are walking in the city center and come across homeless people lying in an underpass. Newspapers are their only protection from the cold and wind. You don’t need much empathy to understand that they are cold, hungry, and often not healthy. They are threatened by things we don’t even notice. A mild cold can turn into life-threatening pneumonia, a small wound into dangerous gangrene. Immunologists have a term for this condition—immunodeficiency, which is caused, in contrast, by too passive or exhausted immune cells. Some could call it callousness or neglect.

A well-functioning immune system is like a smart home—it can recognize and eliminate a dangerous enemy, tolerate friends or completely ignore those who do not bother us. And most importantly—it remembers. So, why not to learn from immunity how to act against current social pathologies?

## Cells with the power of choice

Now, how should an immunity-based society look like? Building it like a military or police academy would be an oversimplification and a misconception. While the immune system is indeed capable of eliminating pathogens and infected or transformed cells like a well-trained army or police force, it also sustains the daily upkeep of our tissues. In short, it maintains homeostasis—a stable internal environment conducive to life and health.

**Figure 1:**
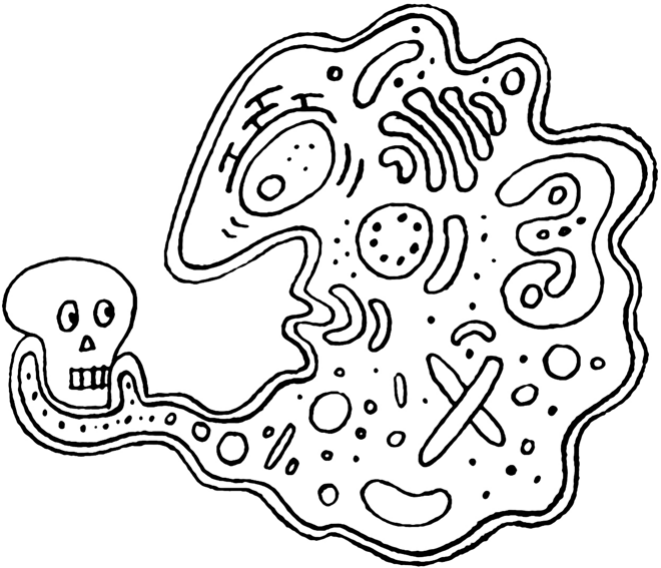
Cell Hamlet by Fero Jablonovský. With permission from the artist.

The immune system is therefore more akin to a whole society than just an army. An immune cell can be a soldier, a repairman, a cleaning lady, a police officer, and so on, depending on the need. Every moment, each immune cell decides whether to fight, clean up or just do nothing (Fig. 1). If, for instance, a T-cell receptor recognizes a foreign peptide bound to the major histocompatibility complex, MHC, on any other mate cell; and simultaneously receives a signal from resident dendritic cells indicating damage in the vicinity, T cell decides to fight—like a police officer noticing shop display shards on a sidewalk. Otherwise, it remains peaceful.

Not only immune cells communicate via cytokines with each other, but also with endothelial, nervous and many other cells; and based on this information make decisions. This is the first lesson immunity teaches us: communicate and make decisions. It is thus no wonder that every dictator suppresses free communication and imposes will by force.

“This is the first lesson immunity teaches us: communicate and make decisions.”

Another equally important lesson is to train memory. As one of Peter Hammill’s songs go: “There’s pain in remembrance, but we must learn not to forget.” Our common memory is preserved in books, paintings, plays, films, or musical works stored in libraries, galleries, theaters, operas, or philharmonics and now on the internet. In addition, human memory is acquired through scientific and philosophical discoveries captured in textbooks, scientific and philosophical journals, and manifested in technologies and social relationships. Every smartphone, every law is an embodiment of our common memory; just as an antibody is an embodiment of immunological memory. Institutions that shape our memory are the bone marrow of society. That’s why every enemy of an open and democratic society attacks them: they burn books, ban films, persecute scientists, and hijack philosophy.

“Every smartphone, every law is an embodiment of our common memory; just as an antibody is an embodiment of immunological memory.”

Then there is education. Immunity has developed a strict but effective schooling system. Immune cells emerging from the bone marrow travel to the tissues where they are needed. However, many of them, specifically T cells, first head to the thymus, the university of the immune system. Here, T cells undergo positive and negative selections. Cells that do not even recognize our own MHC molecules—and are thus useless for defense—fail the former. Cells that recognize our own peptides bound to MHC—and are thus dangerous—do not pass the latter. In a well-functioning immune system, no cell leaves the thymus that does nothing or harm (Brezina et al, 2022; Klein et al, 2014). Similarly, our education system strives to raise people who contribute to a healthy society and do not become dangerous. That is why schools and universities are the next target of the enemies of freedom and democracy.

Attacks on the past—memory—and the future—education—culminate in the capture of parliament and government and the corruption of courts, the three pillars of liberal democracy. This indicates the outbreak of a life-threatening disease. If other institutions are functional, there is hope for recovery, but if the thymus fails to fulfill its function, if the bone marrow is attacked by disease, the prognosis worsens.

“Attacks on the past—memory—and the future—education—culminate in the capture of parliament and government and the corruption of courts, the three pillars of liberal democracy.”

Therefore, another piece of wisdom we should adopt from immunity is to care for functional institutions. Immune cells are endowed with the serine/threonine protein kinase mTOR, mechanistic target of rapamycin, a molecule that regulates their functions and survival in response to external circumstances (Xu et al, 2012). When, for example, energy is limited, immune cells begin recycling proteins and fatty acids to generate the energy needed to maintain functionality.

“… another piece of wisdom we should adopt from immunity is to care for functional institutions.”

We should have a similar system constantly activated in society. If a government emerges that deliberately or out of incompetence neglects cultural or academic institutions, we must stand up for them as one. But this means that we have to trust these institutions. Research during the COVID-19 pandemic showed that trust in science and institutions was the strongest predictor of public health protection (Mousoulidou et al, 2023a; Mousoulidou et al, 2023b). Of course, this condition will hold more probable if institutions consistently make the right decisions.

Just as the quality of institutions depends on the individuals employed within them, the quality of the immune system depends on the diverse types of immune cells that compose it. On social media, I recently came across the quiz titled “Which Immune Cell Are You?” It told me I was a B cell—I do not know why, because sometimes I feel more like an old macrophage. Either way, the question is: What kind of immune cell should each member of society be to ensure a well-balanced immune system? I think this quiz question is somewhat suggestive of determinism. The names of different types of immune cells are human inventions, and cells themselves care little about what we call them – after all, they can change and substitute for one another.

Here we come to the final lesson from immunity—the teacher of life: Let us be like pluripotent stem cells, cells with the power of choice, from which all types of immune cells arise and which can renew the tissues and organs. In this way, we will be capable of creating, forming and renewing our society, to be smart, safe and sustainable.

## Supplementary information

Peer Review File
