# Supplementary material for: Immunitas magistra vitae: The time has come to replace humanity’s teacher of life
Source: EMBO Rep. 2025 Oct 27;26(22):5361–4. doi: 10.1038/s44319-025-00592-5 (PMC12635277; doi:10.1038/s44319-025-00592-5)
Supplement: Supplementary file 1 — Peer Review File [file 44319_2025_592_MOESM1_ESM.pdf]

EMBO reports

TITLE: Imunitas magistra vitae SUBTITLE: The time has come to replace humanity's teacher of life

Vladimir Leksa

Corresponding author(s): Vladimir Leksa (Vladimir.Leksa@savba.sk)

Review Timeline:

Submission Date:	15th Sep 25
Accepted:	26th Sep 25

Editor: Holger Breithaupt

Transaction Report: This article was editorially reviewed.
